# Transfusion of blood stored for longer periods of time does not alter the reactive hyperemia index in healthy volunteers

**DOI:** 10.1186/cc11055

**Published:** 2012-03-20

**Authors:** A Coppadoro, L Berra, B Yu, C Lei, E Spagnolli, AU Steinbicker, KD Bloch, T Lin, HS Warren, FY Sammy, BO Fernandez, M Feelisch, WH Dzik, CP Stowell, WM Zapol

**Affiliations:** 1 University of Milan-Bicocca, Monza, Italy; 2 Massachusetts General Hospital, Boston, MA, USA; 3 University of Warwick, Coventry, UK

## Introduction

The purpose of this study is to investigate the effects of transfusing human packed red blood cells (PRBC) after prolonged storage, as compared to short storage. Retrospective data suggest that transfusion of PRBC stored for over 2 weeks is associated with increased mortality and morbidity. During storage, PRBC progressively release hemoglobin, which avidly binds nitric oxide (NO). We hypothesized that the NO-mediated hyperemic response following ischemia would be reduced after transfusion of PRBC stored for 40 days.

## Methods

We conducted a cross-over randomized interventional study, enrolling 10 healthy adults. Nine volunteers completed the study; one volunteer could not complete the protocol because of anemia. Each volunteer received 1 unit of 40-day and 1 unit of 3-day stored autologous leukoreduced PRBC, on different study days according to a randomization scheme. Blood withdrawal and reactive hyperemia index (RHI) measurements were performed before and 10 minutes, 1 hour, 2 hours, and 4 hours after transfusion.

## Results

The change of RHI after transfusion of 40-day stored PRBC did not differ as compared to 3-day stored PRBC (*P *= 0.67). Plasma hemoglobin and bilirubin levels were higher after transfusion of 40-day than after 3-day stored PRBC (*P *= 0.02 and 0.001, respectively). Plasma levels of potassium, LDH, haptoglobin, cytokines, as well as blood pressure, did not differ between the two transfusions and remained within the normal range. Plasma nitrite concentrations increased after transfusion of 40-day stored PRBC, but not after transfusion of 3-day stored PRBC (*P *= 0.01).

## Conclusion

Transfusion of 1 unit of autologous PRBC stored for longer periods of time is associated with increased hemolysis, an unchanged RHI and increased levels of plasma nitrite in healthy volunteers.

